# Triple Negative Breast Cancer in Pregnancy and Postpartum: Two Case Reports in Hispanic Women

**DOI:** 10.1155/2015/856931

**Published:** 2015-09-13

**Authors:** Ruchi Upadhyay, Qurat-Ul-Ain Butt, Abraham Hamaoui, Cassandra Henderson, Sydney McCalla, Hamid Gilak

**Affiliations:** ^1^Department of Obstetrics and Gynecology, Lincoln Medical and Mental Health Center, Bronx, NY 10451, USA; ^2^Department of Surgery, Lincoln Medical and Mental Health Center, Bronx, NY 10451, USA

## Abstract

*Objective*. Despite studies suggesting that triple negative breast cancer is more often seen in women of African ancestry, we report here two cases of pregnancy associated triple negative breast cancer in Hispanic women. *Cases*. Case one is a 37-year-old female para 2-0-0-2, who presented with a left breast mass, at 19 weeks of gestation, the biopsy of which reported an invasive ductal carcinoma, found to be triple receptor negative. The patient underwent chemotherapy during the pregnancy and was delivered with a cesarean at 37 weeks for obstetric indication. After delivery, the patient completed her chemotherapy that was followed by radical mastectomy and radiotherapy. Case two is a 28-year-old female para 6-0-1-5, who presented while breast-feeding with signs and symptoms of mastitis, and an engorged and tender right breast, five months postpartum. However, the sonogram revealed a fluid filled cavity. Aspiration and cytology did not reflect an infection and were negative for malignancy. High suspicion and lack of improvement led to biopsy that identified an invasive ductal carcinoma, found to be triple negative. The patient underwent chemotherapy followed by modified radical mastectomy. *Conclusions*. Triple negative breast cancer, during pregnancy or postpartum, poses a unique challenge and requires a multidisciplinary team to optimize treatment for these women.

## 1. Introduction

Pregnancy associated breast cancers (PABC) are the second most pregnancy related malignancy, with cervical cancer being the first [1]. PABC are cancers diagnosed during pregnancy or within one year of delivery [2, 3]. Their incidence has been increasing over the years and the prevalence is expected to continue to rise as more women delay childbearing [4]. The occurrence of breast cancer and pregnancy concomitantly poses a unique challenge and management should involve a multidisciplinary approach including obstetrician, maternal fetal medicine specialist, oncologist, neonatologist, and geneticist [5]. Triple negative breast cancer is found more commonly in African American women. Our paper reports two case reports of triple negative breast cancer in Hispanic women.

## 2. Cases

### 2.1. Case One

**Figure 1:**
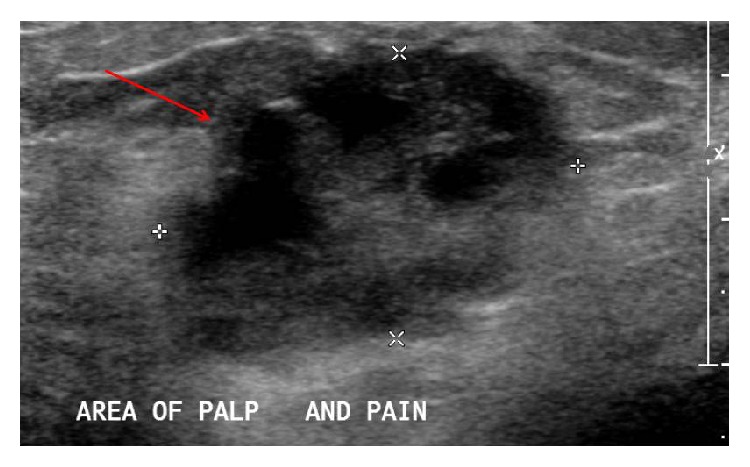
A three-centimeter partially solid mass in the left breast.

**Figure 2:**
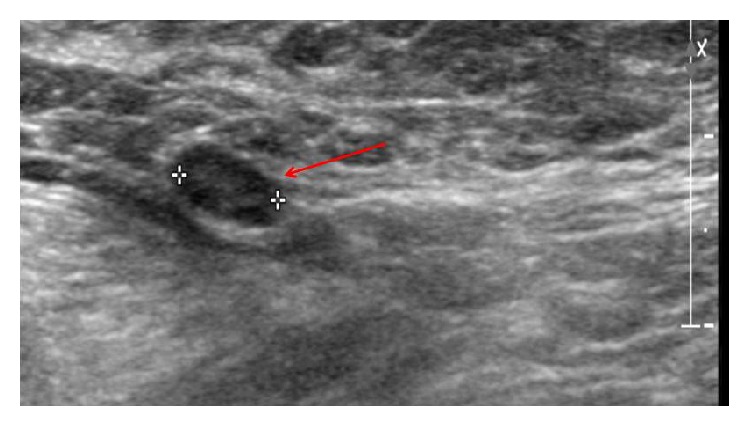
A nine-millimeter indeterminate left axillary lymph node.

A 37-year-old Hispanic woman from Mexico, gravida 2 para 1-0-0-1, presented for initial prenatal visit at 15 weeks of gestation by a first trimester sonogram. Her obstetric history was significant for one term vaginal delivery with gestational diabetes. Her medical and surgical histories were noncontributory. Family history was significant for a second degree relative with breast cancer diagnosed at the age of forty-five. In this pregnancy, she was diagnosed with gestational diabetes with glucose challenge test of 228 mg/dL and was referred to follow-up in high risk clinic. At 19-week visit, she complained of left breast pain for last two weeks. On physical examination, she had a three × four-centimeter firm, mobile, nontender left breast mass, not previously palpated, with no palpable axillary lymph nodes. She had a breast sonogram that revealed a three-centimeter partially solid mass (Figure 1), a nine-millimeter indeterminate left axillary lymph node (Figure 2), Breast Imaging-Reporting and Data System (BI-RADS) category of 4a, low suspicion for malignancy. Ultrasound guided needle biopsy of the mass determined the diagnosis of an invasive ductal carcinoma (IDC), staged at IIB, triple receptor negative estrogen, progesterone, and human epidermal growth factor receptor 2 (HER2). Maternal quadruple screen test was noted for risk of more than 1 : 10 for Down syndrome. Genetic counseling was provided to the patient; patient declined confirmation by amniocentesis.

A multidisciplinary team involving Maternal Fetal Medicine (MFM), breast surgery, oncology, neonatology, and social work was then recruited to discuss and plan the management. Breast surgery and chemotherapy were discussed; she chose chemotherapy, and MFM recommended plan for delivery at 39 weeks. She received three cycles of chemotherapy with doxorubicin and cyclophosphamide during pregnancy. Her gestational diabetes was well controlled with metformin. Fetal surveillance with weekly biophysical profile began at 32 weeks of gestation. She was induced at 37 weeks of gestation for a category two fetal heart tracing and delivered by cesarean delivery for category two tracing remote from delivery. She delivered a live male infant, appropriate for gestational age, with Down syndrome. Postpartum she continued with chemotherapy, followed by left breast mastectomy and radiation therapy. She continues to follow up with Breast Surgery and Oncology Services.

### 2.2. Case Two

A 28-year-old Hispanic woman from Dominican Republic, gravida 7 para 6-0-1-5, presented for follow-up postpartum visit after her sixth delivery. Her obstetric history was significant for previous five vaginal deliveries including one intrauterine fetal demise (at 38 weeks with polyhydramnios) and last pregnancy with gestational diabetes and polyhydramnios. Her surgical and family histories were noncontributory. She continued to follow up in postpartum clinic for routine follow-up and contraception. She was breast-feeding for five months. At five months, she complained of pain in the right breast that is progressively increasing in size and tender for the last two months. She reported using warm compresses that did not help her. She also reported fever and chills off and on. On physical examination, a ten × ten-centimeter mass was palpated on the right breast. She was referred to breast surgery department.

**Figure 3:**
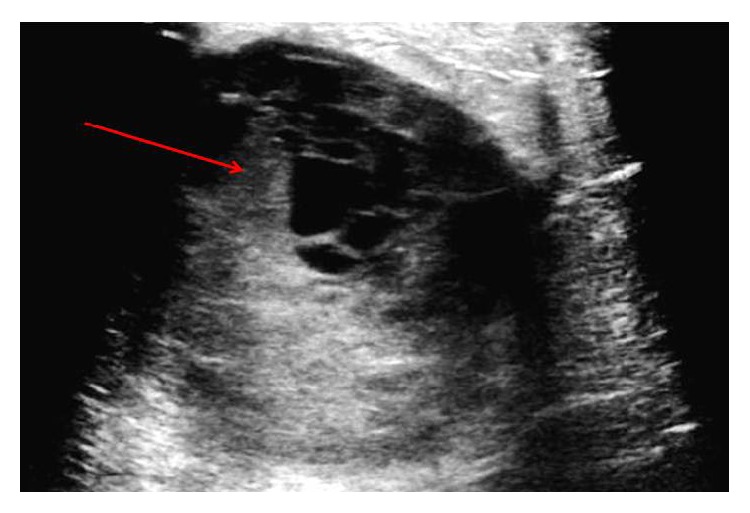
A seven-centimeter fluid lobulated lesion with septations and hypoechoic areas.

Her right breast was aspirated, at bedside at the first breast clinic visit, with serosanguineous discharge that was sent for cultures and cytology, and she was started on antibiotics for mastitis. Breast ultrasound revealed a seven-centimeter lobulated lesion—hypoechoic, fluid filled with septation, likely forming abscess, BI-RADS of 3, probably benign (Figure 3). At follow-up visit, her symptoms did not resolve, physical examination was remarkable for grossly enlarged right breast that was firm and tender with a small erythematous area, and cultures did not grow any bacteria and cytology was benign. Fluid aspiration was repeated at bedside, and antibiotics were changed due to concerns for resistance. Cytology result was negative again, with no resolution of symptoms. Decision was made to proceed with incision and drainage in the operating room. Serous fluid was evacuated in the operating room, and pathology reported IDC, triple receptor negative, estrogen, progesterone, and HER2, stage IIIC. She continued with neoadjuvant chemotherapy with four cycles of dose-dense doxorubicin and cyclophosphamide and paclitaxel, followed by right mastectomy, another cycle of chemotherapy with carboplatin and gemcitabine. She continues to follow up with Breast Surgery and Oncology Services.

## 3. Discussion

Pregnancy favors processes that promote tumor progression including “intense modifications in cell proliferation and survival” and “tissue angiogenesis and remodeling” [2]. Physiological changes in breast during pregnancy, including engorgement, hypertrophy, nodularity, and discharge, make the diagnosis difficult and delayed, leading to poorer prognosis compared to nonpregnant patients [5]. Estrogen and progesterone are known mitogens for the breast tissue and there are theories that suggest the role of these hormones to promote malignant cells in pregnancy [6, 7]. Tumors occurring during pregnancy and those occurring postpartum are considered as two subgroups of PABC, as the former group has a poorer clinical outcome [2]. Studies have shown a higher incidence of cancer-associated death in women with PABC compared with non-PABC tumor [8].

Triple negative breast cancers (TNBC) account for more than 20% of cancers worldwide [9]. They are characterized by lack of estrogen and progesterone receptors and HER2 expression [10] which is attributed to progression of tumor [11]. This type of breast cancers has poorer prognosis compared to any other type as there are fewer therapeutic options [12]. TNBC is associated with higher recurrences and poor survival [11, 13] and even the response to chemotherapy differs from other types of breast cancers [14]. TNBC are more common in African American women [11]. The higher prevalence of TNBC with poor prognosis may be attributed to the high grade and advanced disease at diagnosis along with socioeconomic factors such as deficient health education and health awareness and lack of access to quality care [11].

In both pregnant and nonpregnant patients, the breast cancer presents as a painless and palpable mass [15]. A thorough breast examination must be performed at first prenatal visit and a high degree of clinical suspicion is warranted when a mass is present for more than two weeks and breast ultrasound is the preferred imaging modality for evaluation of a breast mass during pregnancy [5]. Mammography is also used commonly; however, its sensitivity is lowered by the increased density of breast during pregnancy [16]. Both fine needle aspiration and core needle biopsy can be used for evaluation; however, the latter is preferred as it provides information on histology, hormone receptor status, and HER2 analysis [5]. Chest X-ray, ultrasound of the liver, and bone MRI without contrast are recommended to investigate the metastasis of breast cancer [17]. A complete evaluation including laboratories studies and echocardiogram should be considered [16]. There is limited data on the role of the sentinel node biopsy during pregnancy and it is considered controversial [18].

Treatment of pregnant women should not be delayed [19] and a multidisciplinary team approach is warranted to manage these patients throughout pregnancy and postpartum. Both surgery and chemotherapy can be offered to patient. Surgery may be performed during any trimester of pregnancy [20]. Chemotherapy may be used during the second and third trimesters of pregnancy [21]. Hormone therapy and radiation are avoided during pregnancy. Studies have contradictory statements on the overall survival of patients with PABC, ranging from shorter overall survival [22, 23] to similar overall survival [24] when compared to nonpregnant patients. Studies however agree on the minimal effects on fetal outcomes of pregnant women receiving chemotherapy during pregnancy [25, 26] and favor full term over a preterm delivery. These aspects should be considered when counseling the patients.

As triple negative breast cancers and pregnancy associated breast cancers have poor prognosis and diagnostic delays may occur in pregnancy due to effects of pregnancy related hormones, increased awareness and high degree of clinical suspicion among obstetricians and gynecologist may help in optimizing treatment for these women. Future studies are also needed to carefully consider the racial differences and screening programs available for minority populations.
